# Correction: Dissociable mappings of tonic and phasic pupillary features onto cognitive processes involved in mental arithmetic

**DOI:** 10.1371/journal.pone.0256798

**Published:** 2021-08-24

**Authors:** The PLOS ONE Editors

The original Competing Interests statement is incomplete. An updated Competing Interests statement is as follows: SMT is a former employee of Senseye, Inc. (2015–2016) and is named as a co-inventor on a patent/patent application(s) related to eye movement and mental state (assignee, Senseye, Inc). SMT does not consider this a potential competing interest. The other authors have declared that no competing interests exist.

## References

[1] Cohen Hoffing RA, Lauharatanahirun N, Forster DE, Garcia JO, Vettel JM, Thurman SM (2020) Dissociable mappings of tonic and phasic pupillary features onto cognitive processes involved in mental arithmetic. PLoS ONE 15(3): e0230517. 10.1371/journal.pone.0230517 32203562 PMC7089555

