# Supplementary material for: Dissociable mappings of tonic and phasic pupillary features onto cognitive processes involved in mental arithmetic
Source: PLoS One. 2020 Mar 23;15(3):e0230517. doi: 10.1371/journal.pone.0230517 (PMC7089555; doi:10.1371/journal.pone.0230517)
Supplement: S1 Data — (DOCX) [file pone.0230517.s001.docx]

Raw pupil data, as well as trial and session level behavioral data is available at <https://osf.io/cs826/?view_only=f29847cad2f84345a223032377d1d5f8>.
