# Supplementary material for: Dissociable mappings of tonic and phasic pupillary features onto cognitive processes involved in mental arithmetic
Source: PLoS One. 2020 Mar 23;15(3):e0230517. doi: 10.1371/journal.pone.0230517 (PMC7089555; doi:10.1371/journal.pone.0230517)
Supplement: Attachment — Submitted filename: Response to Reviewers.docx [file pone.0230517.s002.docx]

**Reviewer comments and responses:**

We thank the editors for their insightful comments and have provided responses as well as accompanying changes to the manuscript, as noted in bold and by line number, in order in which it appears in the text. Additionally, we have made minor grammatical changes throughout the text. We believe that these changes have improved the clarity of the text by providing additional context and discussion that we hope has resulted in an improved manuscript suitable for publication.

***1a. Editor:*** *As another point, you're taking session into account as a random slope. This implies that you're not interested in how behavior and pupil responses change systematically over time, because those effects will be captured by the by-session random slope. But why not? What is the theoretical motivation behind your statistical models? This point is related to that of Reviewer 1, who would like to see more methodological details.*

**1b.** **Response:** Here we respond to reviewers’ questions and include additional information about the methodology on line (95) and below in **bold**.

The Cognitive Resilience and Sleep History data set (CRASH) was collected to further our understanding of the impact of sleep on cognitive performance. This data set and size, made available to us, was optimized for naturalistic sleep variability with subjects over a long period of time. Specifically, we sought to understand how sleep history modulates the relationship between physiology and behavioral variability. In doing so, we conceptualized sleep loss as a stressor which would impact performance on various tasks known to be influenced by stress, such as attention (psychomotor vigilance task, dynamic visual attention), emotional reactivity (dot probe task), working memory (visual working memory), math performance (mental arithmetic) and a resting state period. We thank the editor for their insightful comment regarding assumptions built into our analysis. As learning is a type of behavioral variability and may track onto pupil-linked processes, we do not have strong theoretical grounds to exclude this type of variance in the analysis. To address this in our analysis we have decided to exclude the random effect of slope from the multi-level modeling analysis. We note that this does not substantially change the magnitude or direction of the relationship between pupil features and response times, suggesting that, in our data set, pupil features do not reflect any systematic change over time. We have re-calculated the statistics accordingly throughout the manuscript in text, tables and figures.

**Participants**

A total of 33 subjects were recruited to participate in the study and asked to complete 8 total sessions over the course of 16 weeks (**Fig 1A**). **The data presented in this manuscript is part of a large-scale, longitudinal study protocol to understand how natural sleep history modulates the relationship between physiology and performance [23]. During the course of the study participants completed daily sleep dairies and wore actigraphy devices. We collected bi-weekly neuroimaging data (i.e. EEG, MRI, fMRI), peripheral physiology (ECG), and eye tracking data while participants completed a battery of tasks in the following order: resting state, psychomotor vigilance, visual working memory, dynamic visual attention, modular arithmetic and dot probe tasks. Each session lasted approximately 120 minutes. The analyses presented in this paper specifically addressed the relationship between pupil size modulations and performance in the arithmetic task.**

Our analysis was restricted to subjects that ended up completing 5 or more sessions, providing a sample size of (N=30, 14 females, 221 total sessions). All procedures were approved by the UCSB Institutional Review Board and the accredited Institutional Review Board at US Combat Capabilities Development Command Army Research Laboratory (ARL) in compliance with the ARL Human Research Protection Program (32 Code of Federal Regulations 219 and Department of Defense Instruction 3216.01).

**Modular arithmetic task**

**In the modular arithmetic task participants completed 40 trials consisting of math statements [10, 11] (total trials: M= 294.66, SD = 35.60) (Fig 1B)** in which participants were asked to subtract the two numbers and respond true if the remainder was divisible by the modulo (i.e. 7), and false if not. Difficulty was manipulated by including subtractions that involved the carrying operation (i.e. 71 ≅ 43 (mod 8)). Stimuli were presented for 8 seconds or until a response was made, whichever occurred first. Feedback was given to participants in the form of a green cross for correct or red cross for incorrect trials, and the cross remained on the screen for 2 seconds. The inter-stimulus-interval consisted of a blank screen randomly presented for 4.5-13.75 seconds.

***2a. Reviewer 1:*** *First, I think a bit more information is needed in the methods, or aspects of the methods just need to be a bit clearer. For example, it looks like participants performed a battery of other tasks in each session and perhaps this was associated with a sleep study. Is that correct?* *A bit more info on when the math task occurred in the battery and the overall reason for collecting the current data is needed. What is the rationale for N = 33. Is this based on an a priori power analysis? Additionally, it looks like participants completed 40 trials per session (8 total sessions) for 320 total trials per participant. Is that correct? Please be sure to really spell out the full methods in detail so that the reader knows what is going on rather than trying to piece it together throughout the methods.*

***2b. Response:*** This comment contained suggestions that were similar to the editors previous comment in 1a and is also addressed by response 1b.

***3a. Reviewer 1:*** *Finally, please provide a bit more information on the mixed effects models. Perhaps include the overall model equations so that the reader knows exactly what effects are in the models.*

**3b. Response:** To address this we now include in methods (**bold;** line 186) the generalized form of the mixed effects model equation that was applied to both trial- and session-level data as detailed below.

**The generalized form of the statistical equation is presented below:**

$\boldsymbol{y}_{\boldsymbol{it}}\boldsymbol{=}\left( \boldsymbol{\beta}_{\boldsymbol{0}}\boldsymbol{+}\boldsymbol{u}_{\boldsymbol{0}\boldsymbol{i}} \right)\boldsymbol{+}\boldsymbol{\beta}_{\boldsymbol{1}}\boldsymbol{x}_{\boldsymbol{1}\boldsymbol{ij}}\boldsymbol{+}\boldsymbol{\beta}_{\boldsymbol{2}}\boldsymbol{x}_{\boldsymbol{2}\boldsymbol{ij}}\boldsymbol{+} \boldsymbol{\beta}_{\boldsymbol{3}}\boldsymbol{x}_{\boldsymbol{3}\boldsymbol{ij}}\boldsymbol{+}\boldsymbol{e}_{\boldsymbol{ij}}$	**(1)**

**In our analysis the outcome variable** $\boldsymbol{y}_{\boldsymbol{it}}$ **represents behavior (i.e. RT and RT distributions). Each predictor variable in its centered form is represented as** $\boldsymbol{x}_{\boldsymbol{ij}}$**, where *‘i’* represents the individual and *‘j’* represents session number. Here, these predictor variables represent the three pupil features (baseline, peak latency and peak amplitude) that were included in the analysis. The intercept of the regression equation is represented as** $\boldsymbol{\beta}_{\boldsymbol{0}}$**. The fixed effects are represented as** $\boldsymbol{\beta}_{\boldsymbol{1}}$**,**$\boldsymbol{\beta}_{\boldsymbol{2}}$**, and** $\boldsymbol{\beta}_{\boldsymbol{3}}$**,. The random effects** $\boldsymbol{u}_{\boldsymbol{0}\boldsymbol{j}}$ **represents the deviation from the intercept by an individual. The term** $\boldsymbol{e}_{\boldsymbol{ij}}$ **represents the residual error.**

**4a. Editor:** On line 339, you mention that:

> Although one possibility is that peak latency is tracking onto the motor response itself, research has found that even in the absence of an overt response, peak latency is associated with the moment of decision selection (8).

*Such easy dismissal of the motor response is unsatisfying to me. Sure, peak latency *can* reflect the moment of decision, but that does not mean that it actually does in your case. To me, the strength of the peak latency v RT correlation suggests that a motor response is part of what drives this correlation. And it's not just the motor response either: the fact that the display changes at the moment of response means that there is a visual change, causing pupil constriction, which is locked to the response. This is not a problem per se (and nothing can be done about it now, in any case), but it's something that warrants additional discussion.*

**4b. Response:** Thank you for this comment. We now further add to the discussion at line (365) and below in **bold**.

The robust relationship between peak latency and decision latency suggests that this pupil feature captures ongoing decisional processes. The mechanism by which this occurs is in line with the idea that the pupil begins to dilate in response to the onset of task-related processing, and continues to dilate according to mental processing demands. According to this framework, the pupil only begins to constrict once **the decision process is complete**, allowing the latency of the peak to robustly capture this decision making process (7, 8, 33).

In comparison to other studies, the remarkable strength of this relationship in our study may also be due to the fact that response times were long which allowed the trial-level pupil response to evolve over a 4-8s time range**. However, it should be noted that one limitation of this study is that we are unable to distinguish between sub-processes driving peak latency, such as decision formation (cognitive-related) versus decision execution (motor-related). Prior work has provided evidence that perceptual decisions in a binocular rivalry task alone can drive pupillary responses [34], while other research has found that motor related processes involved in making a button press (i.e. decision, preparation, execution) can also drive the pupillary response [35]. While motor related research suggests that peak latency reflects the motor response itself, other work has found that even in the absence of an overt motor response, peak latency is still associated with the moment of decision selection [8] suggesting that the motor response may not solely be responsible for driving the correlation with peak latency in the current study. An additional potential factor is the visual presentation of feedback upon response selection, which coincided with a transient visual change following each trial that could also have induced a pupillary constriction. Since we are unable to parse out these possibilities in the current study, how these sub-processes of decision making interact with each other to influence peak latency of the pupil response remains a topic for future research.**

***5a. Reviewer 1:*** *Second, the finding that error responses have larger amplitudes than correct responses is consistent with prior research demonstrating and error pupillary response as noted in the paper. However, the authors seem to suggest that this is due to the feedback provided. But, it looks like the peak response happens around 5 s which is sooner than when the feedback is provided. Thus, this pupillary response seems consistent with overall error monitoring processes independent of feedback.*

**5b. Response:** We agree with the reviewer’s interpretation that the pupillary response to errors (as presented in Figure 4) is more likely driven by an error monitoring process as evidenced by the interaction we report (larger response for incorrect easy vs. correct easy than for incorrect hard vs. correct hard) despite feedback stimuli being identical across conditions. To address this, we have clarified in the revised manuscript that the effect is independent of feedback in text below (**bold**) which appears in line (401). In this task, an arithmetic statement was presented until a response was made or 8s, after which feedback was immediately provided. To clarify the methodology we have changed Figure 1 to reflect the response contingent feedback presentation by changing the response period icon label from ‘8s/Response’ to ‘8s or Response’.

This result appears consistent with the interpretation of peak amplitude as a mapping of attentional state where increased attention is allocated not only during difficult trials but also during surprising events such as getting feedback that an easy question was answered incorrectly. In this case, attention is allocated to an event that engenders learning such as when a prediction error occurs. In fact, previous work has shown that incorrect trials and surprising trials are associated with increased phasic pupil responses [4, 12]. These studies have linked the noradrenaline system, involved in driving the pupil response, to the systems involved in error monitoring underlying prediction errors and learning events. **In support of this interpretation, the aforementioned interaction effect between difficulty and accuracy on peak amplitude, is independent of feedback stimuli, given that the presentation of feedback stimuli was identical for difficult and easy trials. By contrast, peak latency only showed an effect of question difficulty, where longer peak times were related to a longer RT on hard trials.** This result strengthens our earlier interpretation that peak latency closely tracks the decision-making process.

***6a. Reviewer #2:*** *I have one comment regarding the discussion. The present results suggest that pupil dilation and RT variability are positively correlated. In section “Baseline Pupil and Peak Latency are Associated with RT Distribution”, the authors mention that research on sleep deprivation suggests that lower arousal is linked with greater RT variability. However, in the literature on pupillometry higher levels of arousal are often actually linked to larger pupil dilation, going against the presented results. Therefore, the results of this study go against the current understanding of the relationship between arousal and pupil size, which I think is an interesting finding and could be emphasized more.*

**6b.** **Response:** We thank the editor for their insightful comment and further discuss this discrepancy in the literature (**bold**) and on line 422

**Baseline pupil diameter likely reflects longer timescale state effects on performance**

We found that pre-stimulus baseline pupil size was marginally associated with RT variability on individual trials and was not associated with task-related factors like question difficulty or task accuracy. However, by capitalizing on our longitudinal design, we found that baseline pupil size was associated with variability in the breadth of the RT distribution across biweekly testing sessions. The observed correlation between pre-stimulus baseline and IQR aligns with previous research that indicates baseline pupil may reflect an overall arousal state [9, 19, 28, 39]. While baseline pupil size may not co-vary strongly with task-related factors and momentary performance within our 10-minute testing sessions, we did find that it can vary substantially from week to week in relation to global metrics of performance derived from the response time distribution. In line with this finding, research has demonstrated relationships between baseline pupil size and states like fatigue and mind wandering[18, 40].

**While the relationship between baseline pupil size and performance is consistently reported in the literature, the direction of the relationship varies across studies. Our data showed a positive relationship between baseline and IQR indicating that worse performance (i.e. more variability in RTs) was associated with larger baselines, which is consistent with other studies. For example, Gilzenrat et. al. 2010 found that larger pupil diameter was related to a wider RT distribution and Franklin et al. 2013 reported it was related to increased mind-wandering [41, 42]. By contrast, studies like Unsworth et al. 2016 and van der Brink et al. 2016 reported that larger pupil diameter was related to fewer attentional lapses. This discrepancy has been noted elsewhere [15, 16, 43] and discussions have appealed to task and stimulus differences inducing different arousal states and profiles of pupillary responses. For example, tasks may cause shifts on the Yerkes-Dodson arousal curve (formalized as a quadratic), where participants show performance decrements at either end of the curve [43]. We note another possible methodological contribution to the discrepancies across studies is related to differences in methods of computing the baseline pupil feature. In our study, baseline was calculated by taking the average across the entire 10-minute session, whereas in Unsworth et al. 2016 and Gilzenrat et al. 2010 used pre-stimulus baseline measures and van der Brink et al. 2016 used a sliding window of 50 trials (approximately 40 seconds). Because of the dynamic nature of pupil-linked processes influencing the pupillary response it is possible that the baseline feature is sensitive to the temporal length of data used to extract the feature. Our analysis examined baseline pupil size on two different levels (trial-level and session-level), and our results are consistent with this notion. We found that session-level baseline was related to behavior (i.e. RT variability) but trial-level pre-stimulus baseline was not (i.e. no association with condition), suggesting that baseline over a relatively longer timescale was able to capture lower frequency state-level effects on behavior (e.g. changes that occur from session to session). This underscores the importance that studies should consider the interaction between the timescale of pupillary and behavioral change in order to better understand the underlying processes driving pupil-to-performance relationships.**

**Formatting changes:**

**1a**. **Journal:** Please ensure that your manuscript meets PLOS ONE's style requirements, including those for file naming. The PLOS ONE style templates can be found at <http://www.journals.plos.org/plosone/s/file?id=wjVg/PLOSOne_formatting_sample_main_body.pdf> and <http://www.journals.plos.org/plosone/s/file?id=ba62/PLOSOne_formatting_sample_title_authors_affiliations.pdf>

1b. **Response:** Formatting has been changed to conform to the style templates provided.

2a. **Journal:** Thank you for stating the following in the Acknowledgments Section of your manuscript:

'This research is aligned with the scientific aims of the Human Sciences research funded by the United States Army as both in-house research and through university affiliated research centers.

We note that you have provided funding information that is not currently declared in your Funding Statement. However, funding information should not appear in the Acknowledgments section or other areas of your manuscript. We will only publish funding information present in the Funding Statement section of the online submission form.

Please remove any funding-related text from the manuscript and let us know how you would like to update your Funding Statement. Currently, your Funding Statement reads as follows:

'The authors received no specific funding for this work'

2a. **Response:** We opt to keep the Funding Statement as is and have replaced the funding declaration in the Acknowledgments section (line 489) with the following:

‘This research is aligned with the scientific aims of the Human Sciences under the United States Army and conducted as in-house research and through university affiliated research centers.’

3a. **Journal:** Please include captions for your Supporting Information files at the end of your manuscript, and update any in-text citations to match accordingly. Please see our Supporting Information guidelines for more information: <http://journals.plos.org/plosone/s/supporting-information>.

3b.**Response:** We have included a Supporting Information (line 605) section after the references as written below:

**Supporting information**

Raw pupil data, as well as trial and session level behavioral data is available at <https://osf.io/cs826/?view_only=f29847cad2f84345a223032377d1d5f8>.
