# Supplementary material for: Dissociable mappings of tonic and phasic pupillary features onto cognitive processes involved in mental arithmetic
Source: PLoS One. 2020 Mar 23;15(3):e0230517. doi: 10.1371/journal.pone.0230517 (PMC7089555; doi:10.1371/journal.pone.0230517)
Supplement: Attachment — Submitted filename: Response to Reviewers.docx [file pone.0230517.s003.docx]

**Reviewer comments and responses:**

We thank the editors for allowing us to address this final issue to make the manuscript suitable for publication.

**1a. *Reviewer 2:***  *As far as I understand, on lines 202-204 you mention that difficulty and accuracy were included as covariates in the previously described mixed effects model (with RTs as DV and pupil features as IVs). Later in the results, paragraph at line 255, you report a RM-ANOVA with difficulty and accuracy as IVs, and their effects as covariates are not reported. The RM-ANOVA results are of course valid. But it just felt a bit inconsistent to first introduce these factors as part of the mixed model, but then report the results from a separate ANOVA.*

**1b. Response:** Thank you for pointing out these inconsistencies. We did not include difficulty and accuracy as covariates in the *overall* model, but instead ran separate models to test the effects of condition on RT and pupil features (input as DV’s) in four separate models (one model for RT, and one model for each of the 3 pupil features). We clarify this on lines 198-208 and in bold below.

To assess the relationships among pupil predictors and RT on the single trial-level, we ran a mixed effects model with RT as the outcome variable. Pupil baseline, peak amplitude, and peak latency extracted from every trial were included as fixed effect variables in the model. **To look at effects of condition on RT and pupil features, we ran four separate models with either RT or each of the three pupil features as outcomes. In these models, each trial was dummy coded by difficulty (easy=0, hard=1) and accuracy (correct=0, incorrect=1) and included as fixed effects in the model. In all the aforementioned models,** random effects included a random intercept of subject to account for between-subjects variability. This analysis captured whether a relationship existed between the two task conditions and behavior (RT) and/or pupillary responses (baseline, peak amplitude, latency).

**1c.** **Response:** To address the noted inconsistency regarding reporting an ANOVA instead of a mixed effects model, we instead now report the mixed effects statistics as indicated in bold below and starting in the results section on line 247. We note that the repeated measure ANOVA nomenclature was incorrectly used in the prior draft, and we corrected this error by using the mixed effects modeling nomenclature.

**Peak amplitude shows an interaction with difficulty and accuracy**

Previous findings suggest that difficulty (i.e., Easy, Hard) and accuracy (i.e., Correct, Incorrect; **Fig 3A**) should both influence task-evoked pupil dynamics. Difficult math questions require more intense mental processing than easier questions, and therefore should result in a larger pupil dilation response (i.e. larger peak amplitude) [1, 2, 10, 11]. Further, when feedback is provided in real-time on task performance, negative feedback for incorrect trials should also result in larger pupil dilation due to the arousal response associated with indications of failure [4]. Finally, we might expect an interaction between these two factors, whereby the pupil dilation response is increased specifically for easy questions that were answered incorrectly due to the surprise of getting an easy statement wrong [12].

Here, we evaluate the robustness of these relationships at the trial level, in contrast to a majority of prior work that has examined trial-averaged (session-level) pupil responses. To test differences in pupil features by each condition, **we conducted four separate mixed effects models with RT, peak latency, peak pupil and baseline pupil as outcome variables. Difficulty and accuracy were included as fixed effects and subject was included as a random effect.** First, we examined the influence of task-related factors on response time. **As shown in Fig 3B, there was a significant interaction between difficulty and accuracy (ß=-0.097, SE=0.014, p<0.001) as well as a main effect of both difficulty (ß=0.224, SE=0.010, p<0.001) and accuracy (ß=0.169, SE=0.013, p<0.001).** The interaction effect was driven by smaller RT differences between *incorrect* easy (M=3607ms, SD=1351ms) and hard (M=3828ms, SD=1420ms) by comparison to *correct* easy (M=2748ms, SD=874ms) and hard (M=3481ms, SD=1212ms) trials. In other words, while subjects were slower during hard and incorrect statements, there was an additional cost in RT associated with easy questions answered incorrectly.

Peak amplitude showed a similar pattern of results as response times (**Fig 3C**), indicating **an interaction effect (ß=-0.058, SE=0.017, p<0.001) as well as significant effects of difficulty (ß=0.058, SE=0.012, p<0.001) and accuracy (ß=0.103, SE=0.016, p<0.001).** The peak amplitude interaction was driven by a smaller peak amplitude difference between incorrect easy (M=747.39a.u., SD=447.12a.u.) and hard (M=670.85a.u., SD=395.51a.u.) trials when compared to correct easy (M=421.19a.u., SD=244.94a.u.) and hard (M=511.41a.u., SD=294.14a.u.) trials. The response time cost seen in incorrect easy statements is reflected in peak amplitude, with a larger pupil dilation difference for easy incorrect and easy correct statements by comparison to the difference between hard incorrect and hard correct statements.

Peak latency also showed an interaction effect **(ß=-0.040, SE=0.017, p=0.020) as well as significant effects of difficulty (ß=0.113, SE=0.012, p<0.001) and accuracy (ß=0.190, SE=0.016, p<0.001).** The peak latency interaction was driven by a smaller peak latency difference between incorrect easy (M=5.40s, SD=2.24) and hard (M=5.73s, SD=2.20s) trials when compared to correct easy (M=4.59s, SD=2.06s) and hard (M=5.04s, SD=1.96) trials.

**2a. Editor:** *…I became a confused about the analysis that is described on L255 - 266. You describe this as a repeated measures ANOVA with subject as a random effect on trial-level data. To me, this sounds contradictory: either you conduct a RM-ANOVA on aggregated data, or you conduct a LMER on trial level data. The large degrees of freedom also suggest that maybe something went wrong here. (Specifically, I'm thinking that you may have entered trial-level data into a RM-ANOVA test that does not take this into account.)*

*Could you therefore briefly (but clearly) clarify what kind of statistical test was conducted here, and convince me that this is statistically valid? (I'm not a statistician myself, but I'll do my best and possibly consult someone who is.)*

**2b. Response:** Thank you for the comment. We addressed this concern in the response to Reviewer #2.

**3a. *Reviewer 2:*** *on line 171 you repeated "a better" twice in a row*

**3b. Response:** We have corrected this grammatical error.

**Formatting comments and responses**

**1a. Reviewer:** While revising your submission, please upload your figure files to the Preflight Analysis and Conversion Engine (PACE) digital diagnostic tool, <https://pacev2.apexcovantage.com/>. PACE helps ensure that figures meet PLOS requirements. To use PACE, you must first register as a user. Registration is free. Then, login and navigate to the UPLOAD tab, where you will find detailed instructions on how to use the tool. If you encounter any issues or have any questions when using PACE, please email us at [figures@plos.org](mailto:figures@plos.org). Please note that Supporting Information files do not need this step.

**1b. Response:** We addressed this by using PACE to create the figure files which were uploaded to the website.
